# Supplementary material for: Inter-chromosomal transcription hubs shape the 3D genome architecture of African trypanosomes
Source: Nat Commun. 2024 Dec 23;15:10716. doi: 10.1038/s41467-024-55285-9 (PMC11666725; doi:10.1038/s41467-024-55285-9)
Supplement: Supplementary file 1 — Supplementary Information [file 41467_2024_55285_MOESM1_ESM.pdf]

Inter-chromosomal transcription hubs shape the 3D genome architecture of African trypanosomes

Rabuffo et al.

Supplementary information

Supplementary Tables

Supplementary Table 1. ONT sequencing libraries information.

Replicate	Cell line	Platform	Estimated bases	Data produced	Reads generated	Estimated N50
**BioR1_TechR1**	Wt lister 427	GridION	9.2 Gb	99.53 GB	434.48 k	43.33 kb
**BioR1_TechR2**	Wt lister 427	GridION	3.54 Gb	38.06 GB	139.55 k	49.68 kb
**BioR2_TechR1**	Wt lister 427	PromethION	1.16 Gb	14.37 GB	17.95 k	438.95 kb
**BioR2_TechR2**	Wt lister 427	PromethION	863.05 Mb	9.88 GB	42.29 k	39.79 kb
**BioR2_TechR3**	Wt lister 427	PromethION	5.85 Gb	64.76 GB	342.92 k	48.98 kb
**BioR3_TechR1**	P10	PromethION	11.86 Gb	138.45 GB	381.45 k	89.55 kb

Supplementary Table 2. Sequencing statistics for Micro-C libraries (BioR = biological replicate; TechR = technical replicate).

	TechR1, BioR1	TechR2, BioR1	TechR3, BioR1	TechR1, BioR2	TechR2, BioR2	TechR3, BioR2	TechR1, BioR3	TechR2, BioR3
**Total reads**	25261037	32462980	24713165	37041198	38214510	43914719	115512475	102625990
**Total unmapped reads**	3995106	4030923	4303032	5012643	5156277	5615086	17019343	17194172
**Total mapped**	14178832	19229417	13722240	22173091	22643575	26524174	63174015	55915590
**Total duplicates**	1138469	1807501	3005303	1906352	2048070	2359035	29177780	25497442
**Total no duplicates**	13040363	17421916	10716937	20266739	20595505	24165139	33996235	30418148
**Cis interactions**	10253355	13684823	8548699	15536829	15807050	18382706	24528717	22952715
**Trans interactions**	2787008	3737093	2168238	4729910	4788455	5782433	9467518	7465433
**Cis int. >1kb**	6823990	9763626	5723472	10788591	10499315	12742026	17093054	14144033
**Cis int. >2kb**	5807532	8324817	4892989	9120455	8873271	10711000	14871485	12324841
**Cis int. >10kb**	4167697	5921336	3486252	6489101	6352132	7612237	10879079	9023096
**Cis int. >20kb**	3535582	5013329	2947316	5506230	5408468	6468406	9357929	7760004
**Cis int. >20kb**	2867708	4061629	2381138	4483027	4421335	5277899	7758176	6429487
**Fraction of cis interactions**	0.78628	0.78549	0.79768	0.76662	0.76750	0.76071	0.72151	0.75457
**Fraction of cis >1kb**	0.52330	0.56042	0.53406	0.53233	0.50979	0.52729	0.50279	0.46499
**Fraction of cis >2kb**	0.44535	0.47784	0.45657	0.45002	0.43084	0.44324	0.43745	0.40518
**Fraction of cis >10kb**	0.31960	0.33988	0.32530	0.32018	0.30842	0.31501	0.32001	0.29664
**Fraction of cis >20kb**	0.27113	0.28776	0.27501	0.27169	0.26260	0.26768	0.27526	0.25511
**Fraction of cis >40kb**	0.21991	0.23313	0.22218	0.22120	0.21467	0.21841	0.22821	0.21137
**Fraction of duplicates**	0.08029	0.09400	0.21901	0.08598	0.09045	0.08894	0.46186	0.45600
**Complexity naive**	83501310.07	95772170.35	26563987.84	121447326.33	117506824.25	140135261.36	45122956.49	40751622.29

Supplementary Table 3. Centromeric repeats and their classes. Repeats were identified using the algorithm Tandem Repeat Finder[1] and the best hit was reported for each centromere. In some cases, e.g., centromere of chromosome 9B, there are two best hits for the first and last part of the repeated region. Centromeres were grouped in four classes based on the degree of sequence similarity and formed by: I) centromeres that are located on subtelomeric regions and centromeres of chromosomes 1 and 6; II) centromeres on chromosomes 2 and 7; III) centromeres on chromosomes 4, 5 and 8, characterized by the CIR147 repeat; IV) the centromere on chromosome 3. Alignment of each repeat against the others shows a partial overlap between centromeres of class I and class III.

Centromere on chromosome	Repeat size (bp) and class	Sequence 1	Sequence 2
**1A/1B**	146, **class I**	AATAAGCAATAATACGCAATAATACACATTAA<br>TGCACACATATGCGTGCTTATTCAATAATGAG<br>CAATAATAAGCAATAATAAGCAATAATACGCA<br>ATAATGCACATTAATGCACACATATGCGTGCT<br>TTTGCGATAATGCGCAAT	
**2A/2B**	29, **class II**	TGTTTTATGCACAAAAGCGTGTTATTAAG	
**3A/3B**	120, **class IV**	GATTGGGTAACGCCCTTCCACTGATCACATGC<br>ATTGGTGGCACATCATGGCCCATGTTTCATGGT<br>TATCACCCCTACGGCGCATAATGGTGTGTTATC<br>GCACAAAACCCTGTTACAGTGT	
**4A/4B**	148, **class III**	CTACACTGTTTTTGCACAATCATACGCATAAAC<br>GCGTATTTGACGTGAAAATACGCAATAGTGCA<br>CAATTTGTGAACAAACATGCCACATTGTGCAT<br>GTTATGCAAGAATGCGTGTTTTACACAAAAAC<br>ACTTTACATGTTGCGTTG	
**5A/5B**	147, **class III**	TGCAACACTGTTTTTGCACAATCATACGCATGA<br>ATGCGTATTTGACGTGAAAATACGCAATAGTG<br>CACAATTTGTGAACAAACATGCAACATTGTGC<br>AGGTTGTGCAAGAATGCGTGTTTTACACCAAA<br>ACACCTTACATGTTGCAT	
**6A/6B**	58/136, **class I**	TGTGTAATTATACGCAATAATGTGCAATTTGTG<br>CAATTATACGCAATAATGTGCAATT	AATTATACGCAATAATGTTC<br>ACTTTGTGCAATTATACGCA<br>ATAATGTGCACTTTGTTTAA<br>TTATACGCAATAATGTGCAT<br>TTATACGCAATAACGTGCA<br>ATTTGTTTAATTATACGCAA<br>TAATGCGCACTTTGTTT
**7A/7B**	30, **class II**	TTATTAAGTGTTTTATTTTGAAAAAGCGTG	
**8A/8B**	147, **class III**	AAAACACGCATTCTTGCACAACATGCACAACG<br>TTGCATGTTTGTACACAAAATGTGCACTATTGC<br>GTATTTTCACGTAAAATACGCGTTCATACGTGT<br>GATTGTGCAAAAACAGTGTTGCAATGCAACAT<br>GTAATAACGTGTTTTGGTTT	
**9A**	39, **class I**	AGGCAATAATGCACATTTTAAGTCATAATGTG<br>CAATAAT	
**9B**	39/78, **class I**	AATGTGCAATAATAGTCAATAATATGCACTTT<br>ACGCCATAATGTGCAATAATAGTAAACAATGT<br>GCACTTTACGCCAT	AATGTGCAATAATAGTAAA<br>CAATGTGCACTTTACGCCAT
**10B**	49, **class I**	GTGCAATAATGTTCATTTTACGCAATAATGTTC<br>AATAATA	
**11A**	49, **class I**	GCAATAATGTGTATTTTACGCAATAATGTGCA<br>ATAATGTGCAATAATAG	
**11B**	20/60, **class I**	ATTGCACATTATTGCCTATT	TTATTGCCTATTATTGCACA<br>TTATTGCCTATTATTGCACA<br>TTATTGCCTATTATTGCACA

Supplementary Table 4. Primers for FISH probes.

Target Region	Forward primer (5’-3’)	Reverse primer (5’-3’)
**147bp repeats (CIR147)**	ATGCACAATGTGGCATGTTT	AACACTGTTTTTGCGCAATC
**Spliced Leader region 1**	TGGTATGAGAAGCTCCCAGT	TGTTTGCGTGTGTGTGTCAG
**Spliced Leader region 2**	ATCTGTATAAGCGCGTTGGG	CAGCAGACTTTAAAGCGCCT
**Spliced Leader region 3**	TGTTCTTAACCTGGTTATACCCGCA	GAAGAAGGACGGTTGAGCTGAGTGTA
**Spliced Leader region 4**	TGTGTTCTATATAAAGTTTATCGGCA	ATGGTGAATCGCCATCTGCAACAG
**Spliced Leader region 5**	AGATGGCGATTCACCATTAAGCAT	TTGAAAGCATGGGTGTGTCC

Supplementary Figures and Notes

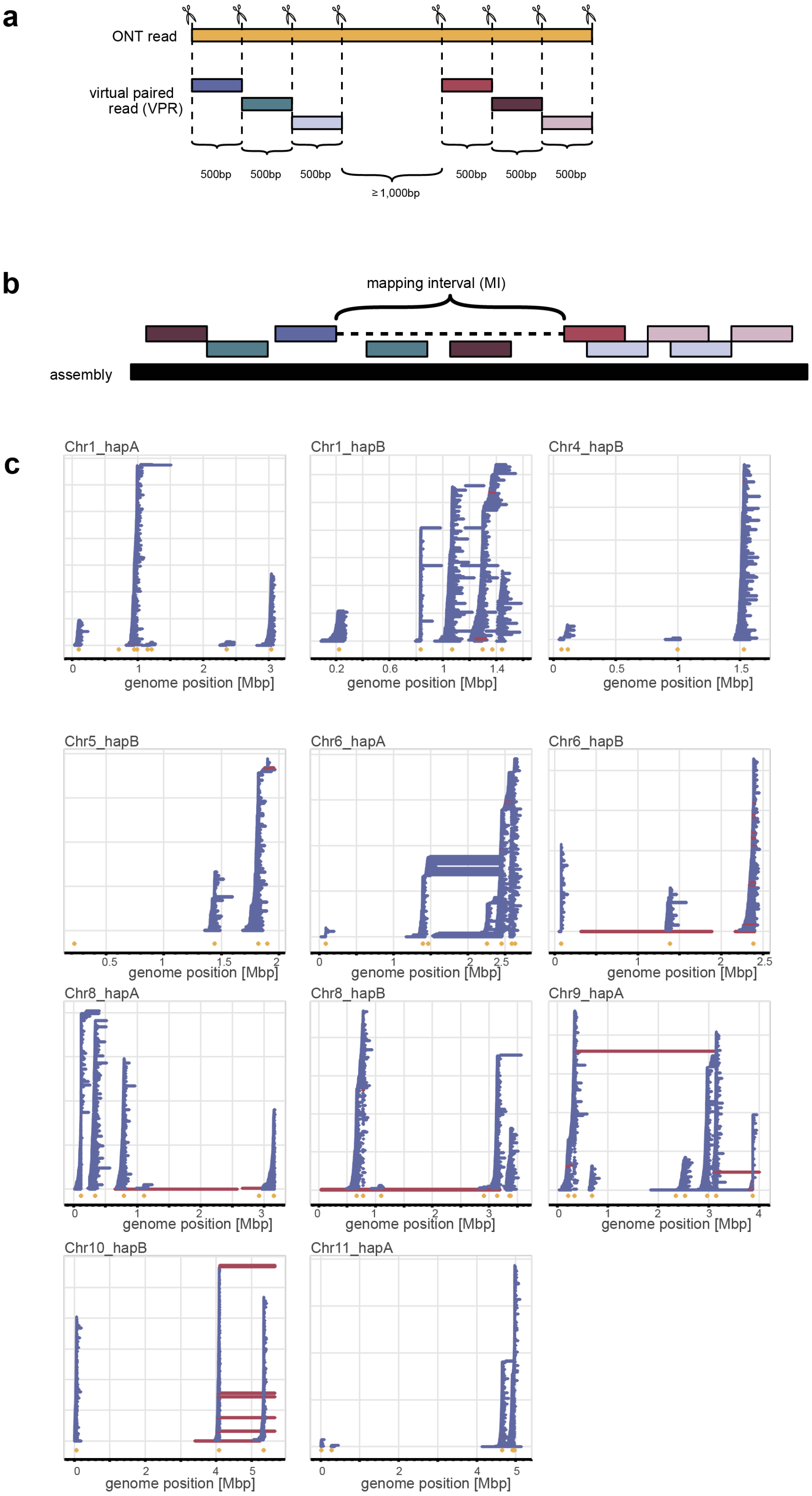

Supplementary Figure 1. Virtual paired reads (VPRs) confirm that small contigs have not been scaffolded in inverse orientation. a An Oxford Nanopore Technologies (ONT) long read is cut into a virtual paired read (VPR). For each read in the virtual pair, three sections are cut out (each section of the VPR is displayed in a different color). **b** The VPRs are aligned on each end of the ONT read the VPR with the highest mapping quality is picked (the leftmost

VPR read on either side of the ONT read in the example). The mapping interval (MI) of the VPR is the distance between the picked reads alignments. **c** All MIs of VPRs that overlap a gap are displayed. Gaps where all overlapping VPRs align to the same strand have been excluded. MIs of VPRs that map to the same strand are drawn in blue, while those mapping to opposite strands are drawn in red. Gaps are drawn as yellow crosses. (See Supplementary Note 1 for more details).

Supplementary Note 1 - Closing gaps and expanding repeats in the assembly

To improve the *T. brucei* genome assembly, our goal was to close gaps and expand collapsed regions without affecting the remaining, well-assembled parts of the genome. An error in BES 2 of the Lister 427 HGAP3_Tb427v10[2] assembly was corrected, resulting in the generation the Tb427v11 assembly. Ultra-long Oxford Nanopore Technologies (ONT) read datasets were generated with GridION and PromethION and used together with ONT reads from Girasol et al.[3] for the following analyses.

The contigs of the Tb427v11 assembly were scaffolded using Hi-C data. Such Hi-C-based scaffolding is known to orient smaller contigs the wrong way around[4]. Such erroneous scaffolds were checked using virtual paired reads (VPRs, see the Methods section in the main text). Supplementary Fig. 1c shows all VPRs with mapping intervals (Mis) that span over gaps, where the gap has at least one VPR mapping to opposite strands. For all gaps, most reads map to the same strand on either side, therefore there was no evidence that a contig has erroneously been reverse-complemented in the Tb427v11 assembly.

Virtual paired reads

First, VPRs were generated from the ONT reads by cutting the three outermost 500 bp on either side from every ONT read that is at least 4 kb in length. These 500-bp-long virtual reads were aligned individually, keeping the piece with the highest mapping quality on either side of the ONT read (Fig. 1c and Supplementary Fig. 1a). The rationale for testing three 500 bp segments on either side is the repetitiveness of the genome: If the outmost ends of an ONT read are located in repetitive regions, aligners cannot determine a unique genomic location of these regions, making the ONT read unusable. Using three segments allows rescuing some ONT reads that would otherwise not be usable.

The MI of a VPR is defined as the distance between the mapping loci of the two reads in the VPR (Supplementary Fig. 1b). The distance deviation (DD) of a VPR is computed as the difference between the distance of the VPRs' mapping loci on the assembly and the distance of the VPR on the ONT read.

Closing gaps

In the Tb427v11 assembly some sequences flanking gaps had been erroneously duplicated to both sides of these gaps. When aligning ONT reads to these regions, the duplicated sequences could only be observed once in the reads (Supplementary Fig. 2a), hence the gaps could not be closed correctly. To identify and correct such cases, VPRs were clustered using a single-linkage approach that joins two VPRs if their MIs overlap and their difference in distance deviation (DD) is below 500 bp (Supplementary Fig. 2b,c). The DD of a VPR is computed as the difference between the distance of the VPRs' mapping loci on the assembly and the distance of the VPR on the ONT read. A positive DD hence indicates that some sequence is missing on the analyzed genome, while a negative DD indicates a superfluous sequence on the assembly. All clusters consisting of five or more reads were kept. A gap was identified as 'duplicated' sequence if 1) a cluster of VPRs with DD $>$ 500 bp and MIs enclosed the gap; and 2) no cluster of VPRs with DD $<$ 200 bp and MIs enclosed the gap (Supplementary Fig. 2d). The sequence covered by all MIs was cut away, followed by another round of gap closing with SAMBA[4].

Next, gaps occurring on both alleles, where sequences flanking the gaps were highly homozygous, were identified. Such gaps in homozygous regions could not be closed by SAMBA. Hence, the alleles of the megabase-sized chromosomes were split into two assemblies. Two read subsets were created by aligning all reads to the whole genome. For subset A, all reads that had their primary alignment on allele B were removed. For subset B, all reads that had their primary alignment on allele A were removed. SAMBA was run two more times, once using read subset A and allele A and once using subset B and allele B.

Expanding repeats

VPRs were used to identify collapsed repeats in the assembly. Specifically, VPR read clusters with a DD < -100 bp that had MIs further than 1 kb from any remaining or closed gap in the assembly were used to call collapsed repeats (Supplementary Fig. 3). To extend these repeats, the identified regions were masked with Ns, turning them into gaps in the assembly. SAMBA was run to close these gaps. For masked regions that could not be filled by SAMBA, the removed sequence was pasted back into the gap.

Assessing the length of newly assembled regions

Newly assembled regions were designated 'correct' (Fig. 1d) if there was a VPR cluster, consisting of five or more reads, with a DD < 5kb and MIs that fully overlapped the region.

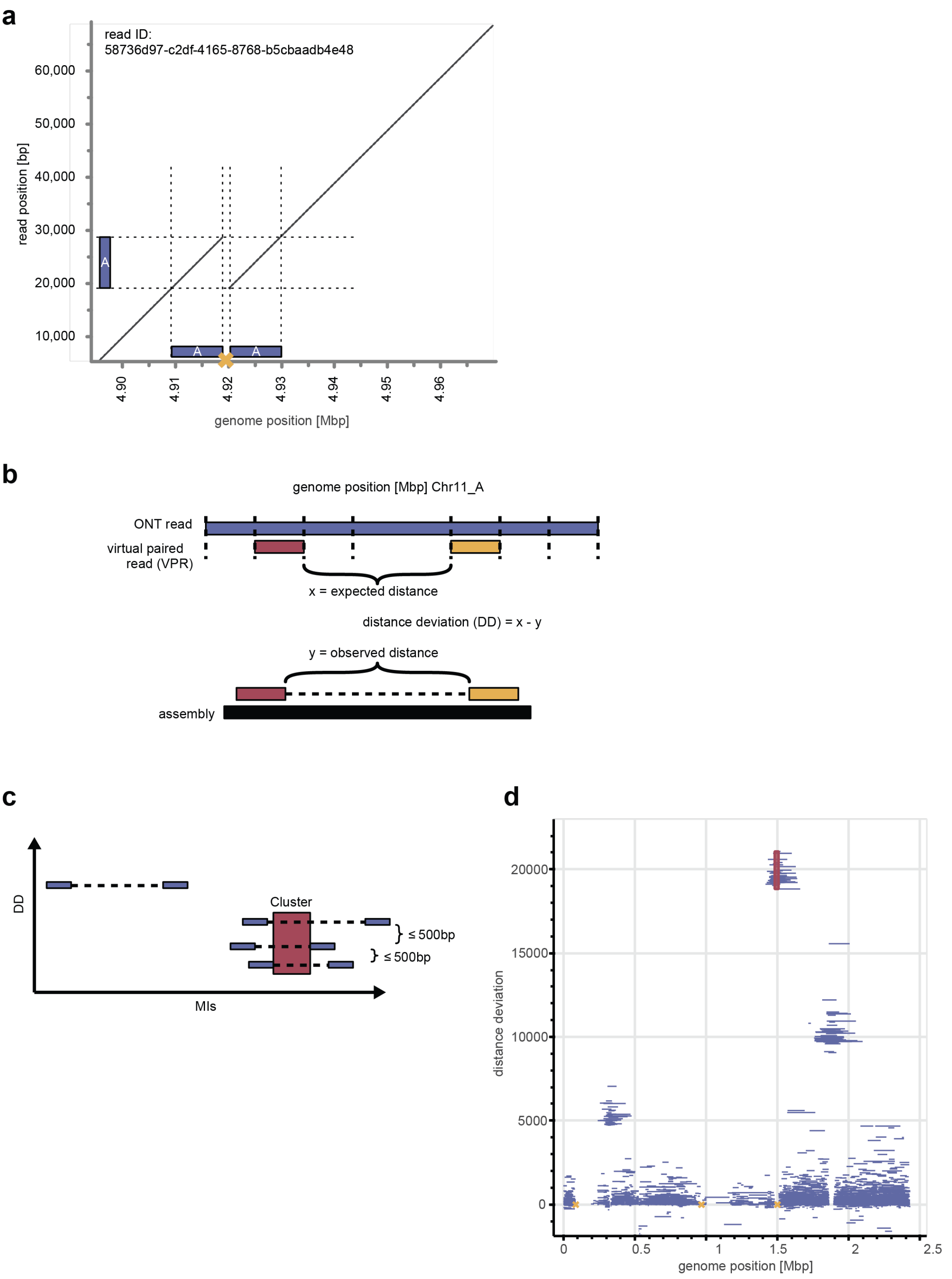

Supplementary Figure 2. **Removing duplicated sequences adjacent to gaps using VPRs. a** The alignment of a read against chromosome 11A of the Tb427v11 assembly displayed as a dot-plot. The blue section labeled 'A' occurs once on the read and twice on the assembly. The position of the gap on the assembly is indicated with a yellow cross. **b** We compute the distance deviation (DD) of VPRs (displayed in red and yellow) by subtracting the length of their MI from the distance of the VPRs on their ONT read. **c** Clusters of VPRs contain reads with overlapping MIs and DD differences of smaller than 500 bp. The cluster width is the common section of all MIs. **d** Shown is chromosome 4A of the Tb427v11 assembly. The MI of VPRs are drawn as blue lines, where the y-position of the line is determined by the DD of the VPR.

Yellow crosses indicate the gaps in the assembly. The third gap is expected to have some duplicated sequence on either side, as can be seen by the cluster (red rectangle) of VPRs that spans the gap. The VPRs with DD of ~5,000 and ~10,000 are not a cluster since they do not overlap a gap.

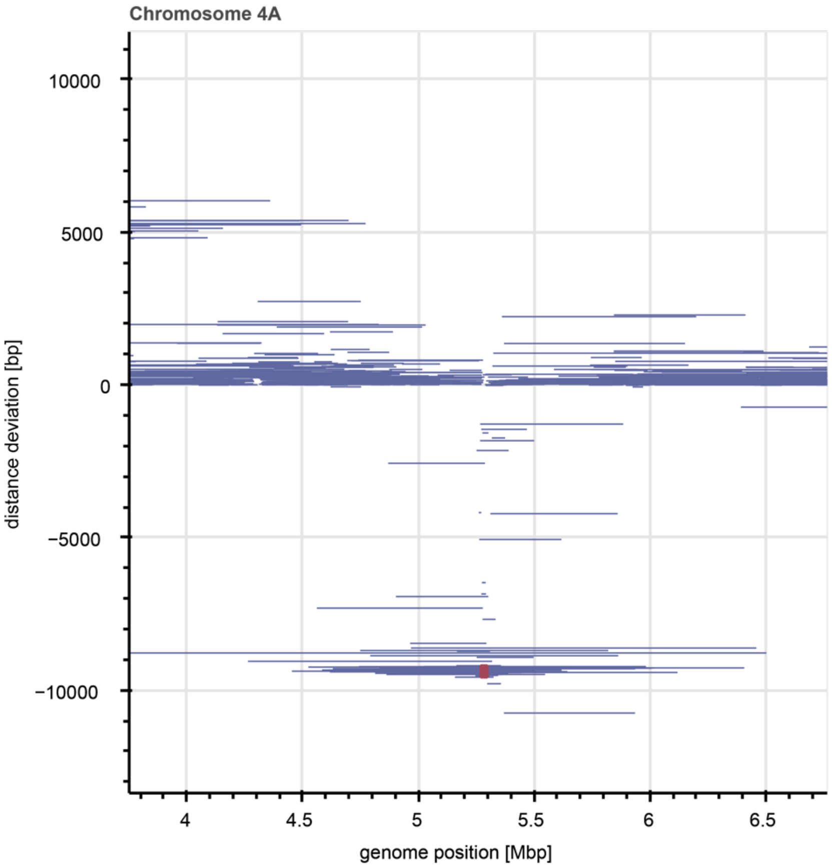

Supplementary Figure 3. **A collapsed repeat on chromosome 4A of the Tb427v11 assembly.** A collapsed repeat on chromosome 4A of the Tb427v11 assembly. MIs of VPRs are drawn as blue lines, where their y-position indicates the DD of the VPR. The red box indicates the cluster.

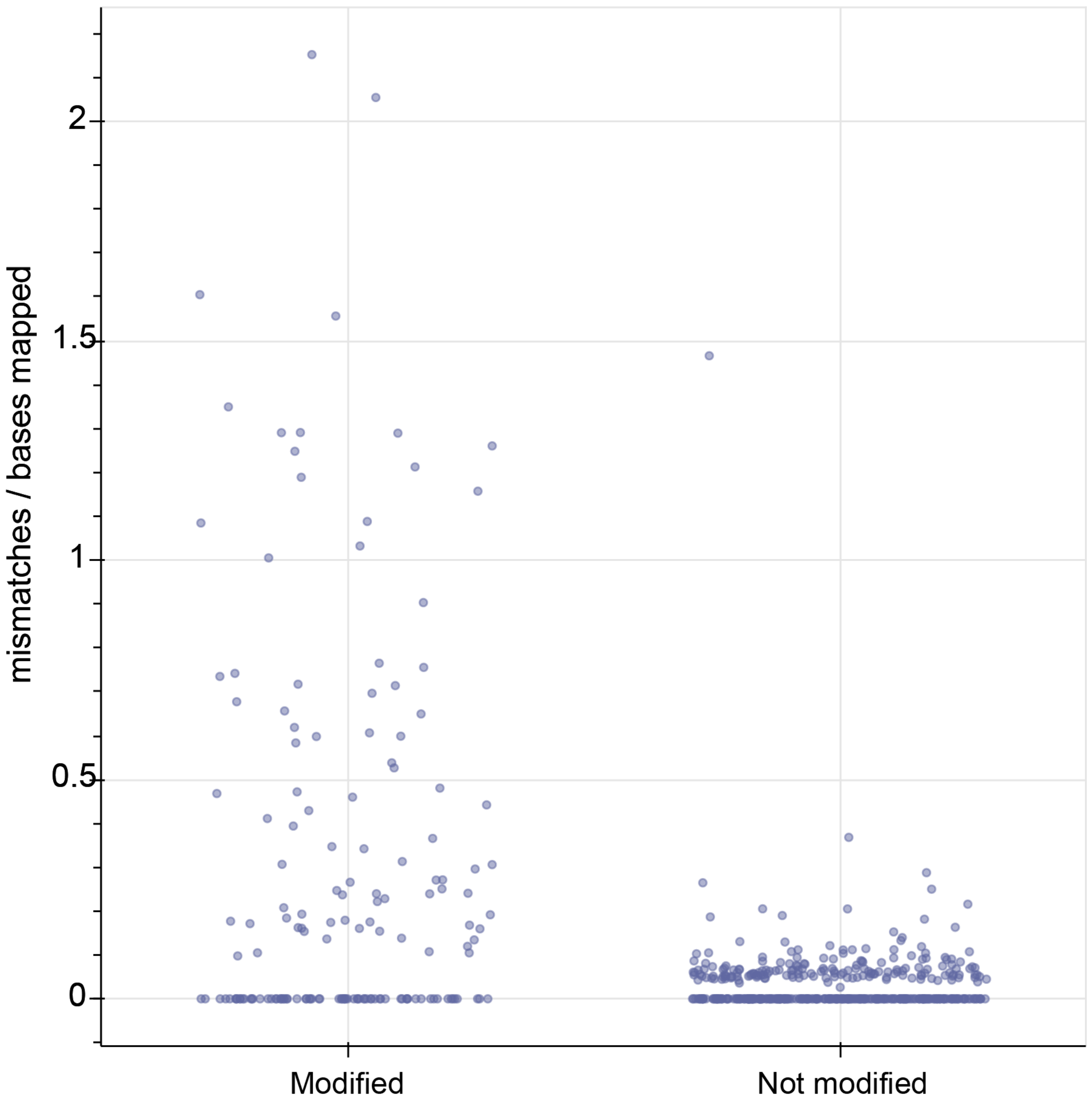

Supplementary Figure 4. **The ratio mismatches over mapped bases for each modified region (n=159) and each non-modified region (n=438).**

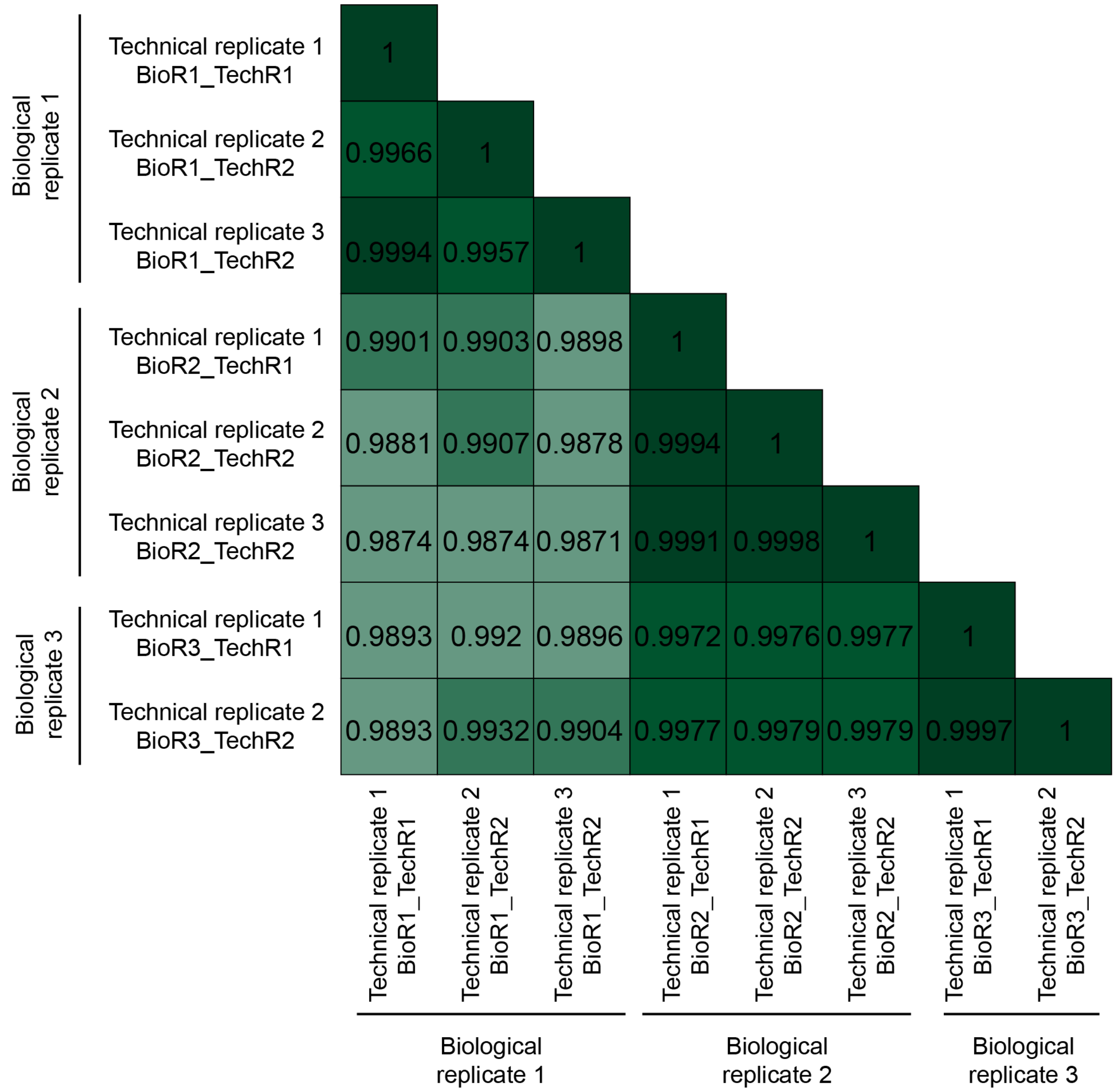

Supplementary Figure 5. **Reproducibility of Micro-C libraries**. Reproducibility between Micro-C sequencing libraries as Stratum-adjusted Correlation Coefficient (SCC) calculated with HiCRep[5] at 100 kb resolution. BioR = Biological replicate; TechR = Technical replicate.

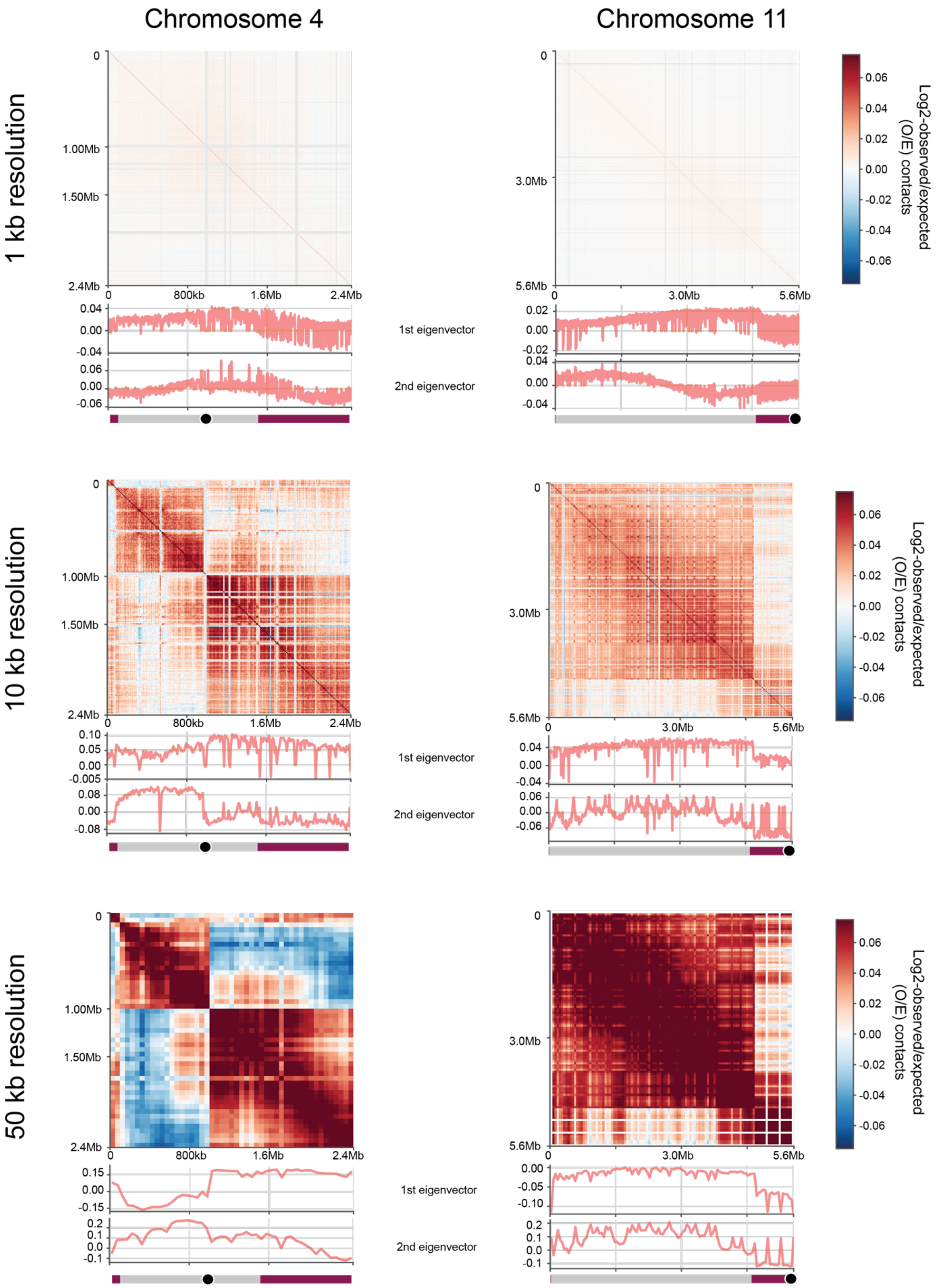

Supplementary Figure 6. Correlation matrices and eigenvector decomposition of chromosomes 4 and 11 at different resolutions. Correlation matrices and eigenvector decomposition were calculated using FAN-C[6] on the Micro-C matrix obtained according to the distiller pipeline at 1, 10 and 50 kb resolution. Below each matrix, first and second eigenvectors

are plotted. Each chromosome is displayed under the eigenvector plots with the core region in grey and subtelomeric regions in dark red; centromeres are represented as black dots.

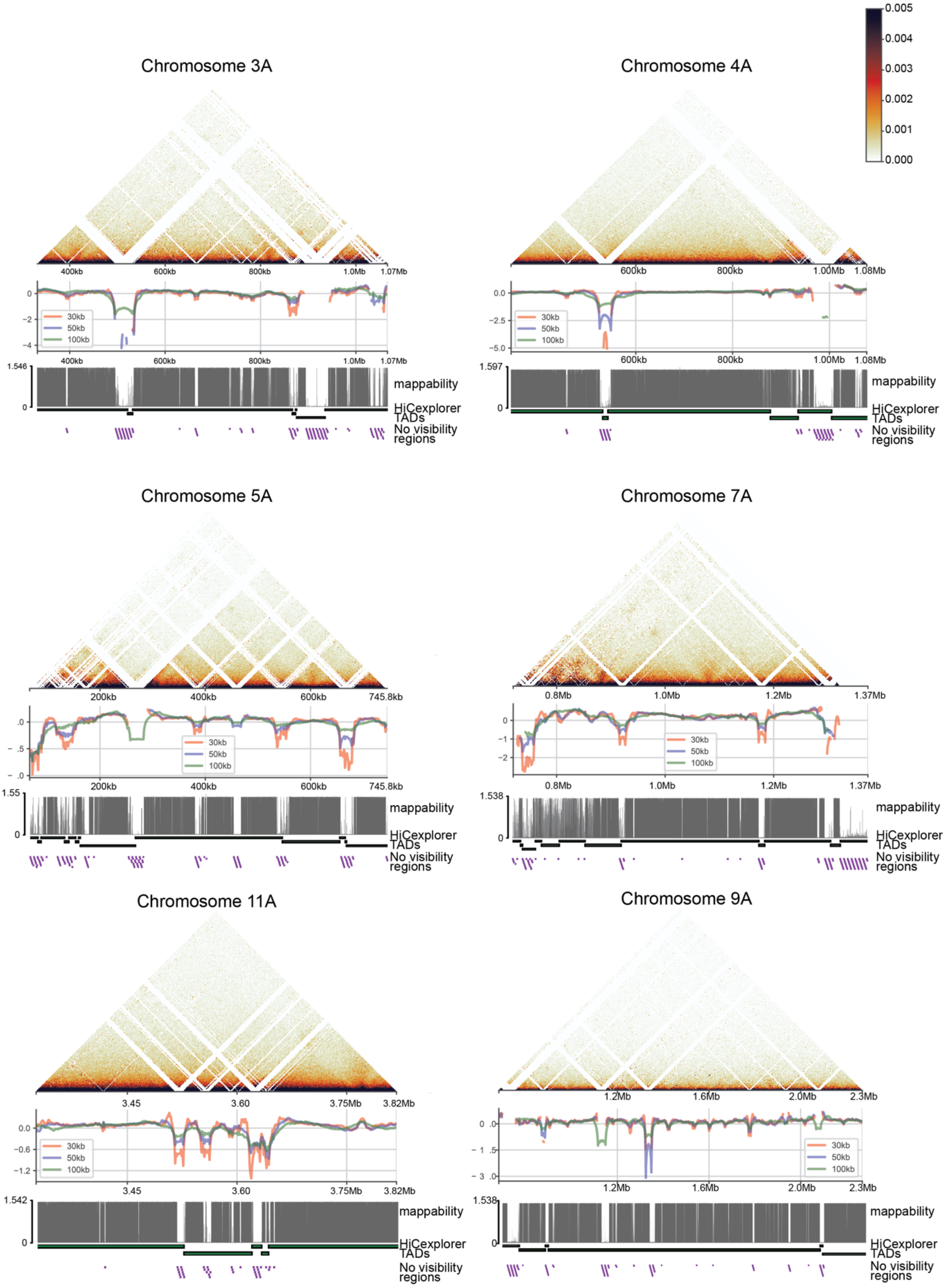

Supplementary Figure 7. **Example of insulation scores calculated by FAN-C.** Insulation scores were calculated using FAN-C[6] on the Micro-C matrix generated using the Distiller pipeline and plotted at 30, 50 and 100kb resolution. Mappability (see Methods), TAD-like domains identified by HiCExplorer[7] and annotated no visibility regions (i.e., regions where signal was removed by IC normalization; for details see Methods) were displayed (bottom).

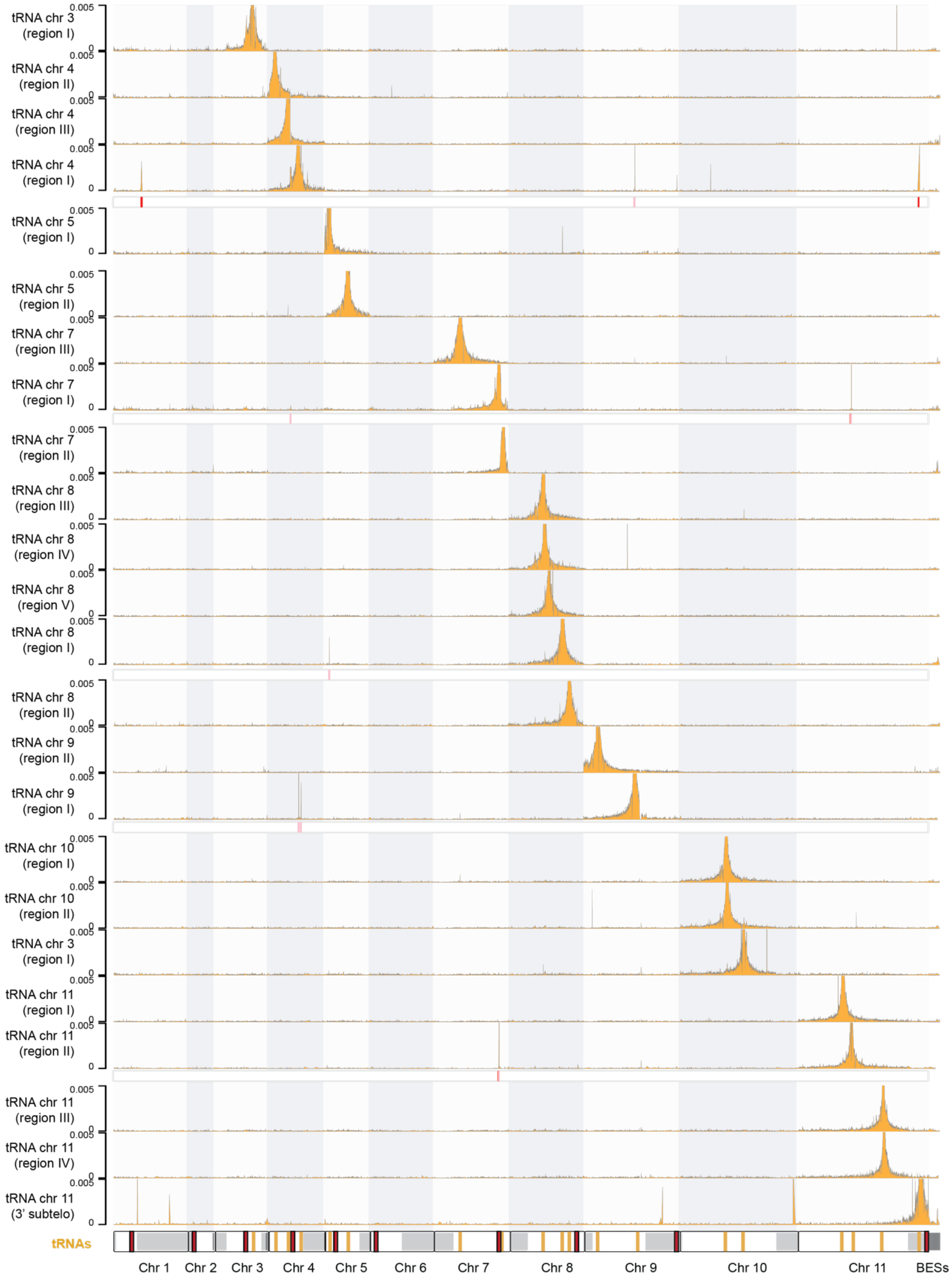

Supplementary Figure 8. Interaction frequencies of tRNA regions against the *T. brucei* genome, haplotype A. Virtual 4C interaction profiles based on Micro-C data (5 kb resolution) showing the interaction frequencies (y-axes) of tRNA regions as points of view with the Tb427 genome version 12, haplotype A. tRNA regions were defined as groups of adjacent tRNA loci separated by less than 10 kb. Each region was identified by the chromosome it belongs to and an additional numeric identifier. Significant interactions of interacting tRNA loci calculated

using a two-sample KS-test were marked below each track (pink: $p<0.05$; red: $p<0.01$). Location of centromeres (red) and tRNA regions (yellow) are displayed below. Each chromosome is displayed with the core region in white and subtelomeric regions in light gray.

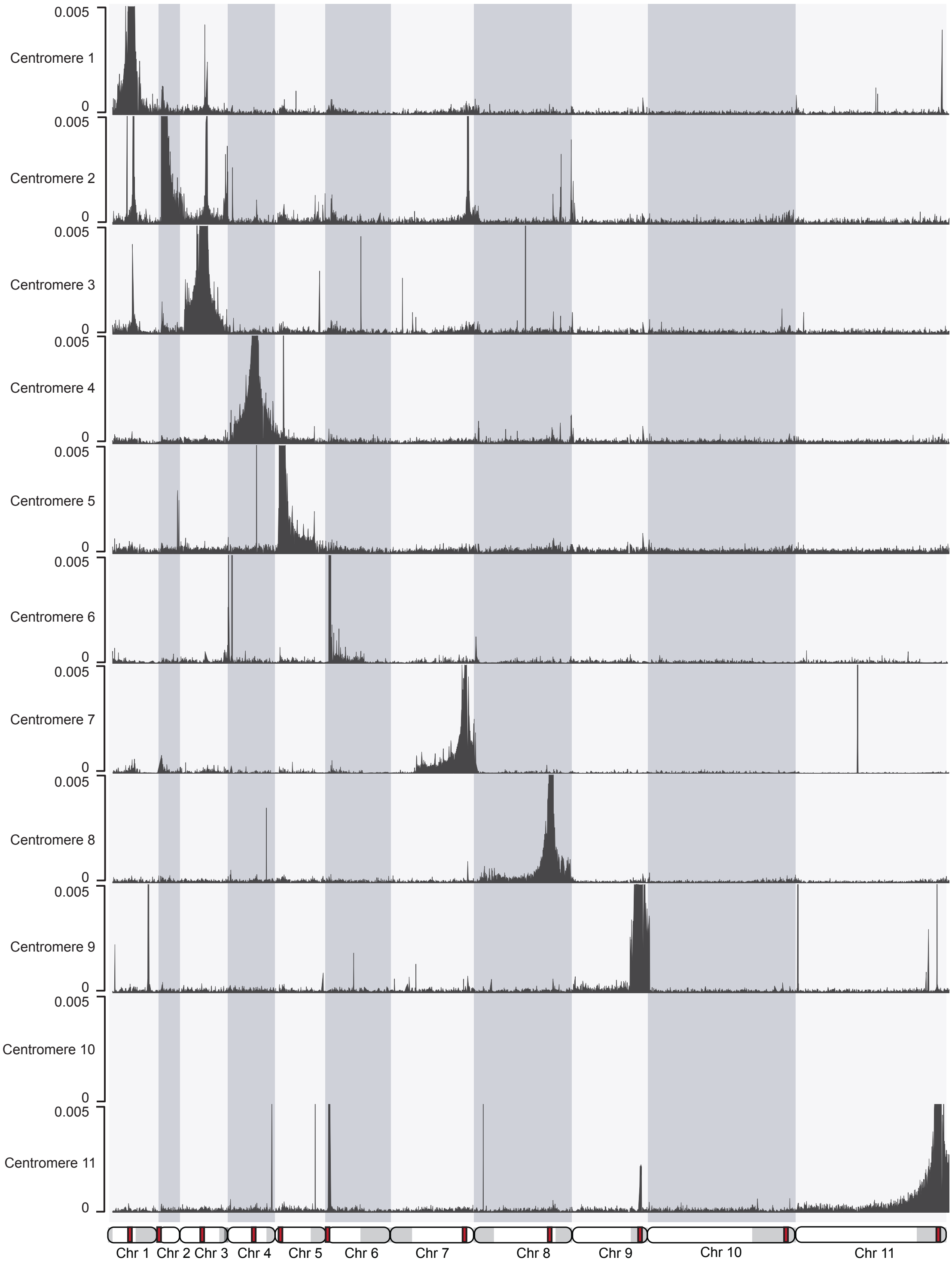

Supplementary Figure 9. Interaction frequencies of regions adjacent (upstream) to centromeres of haplotype B plotted against the entire genome, haplotype B. Virtual 4C interaction profiles based on Micro-C data (10 kb resolution) showing the interaction frequencies (y-axes) of 5 kb regions upstream the bins containing centromeres of genome B as points of view with the Tb427 genome version 12, haplotype B. Bottom: each chromosome is displayed with the core region in white and subtelomeric regions in light gray. Centromeres are displayed as red boxes.

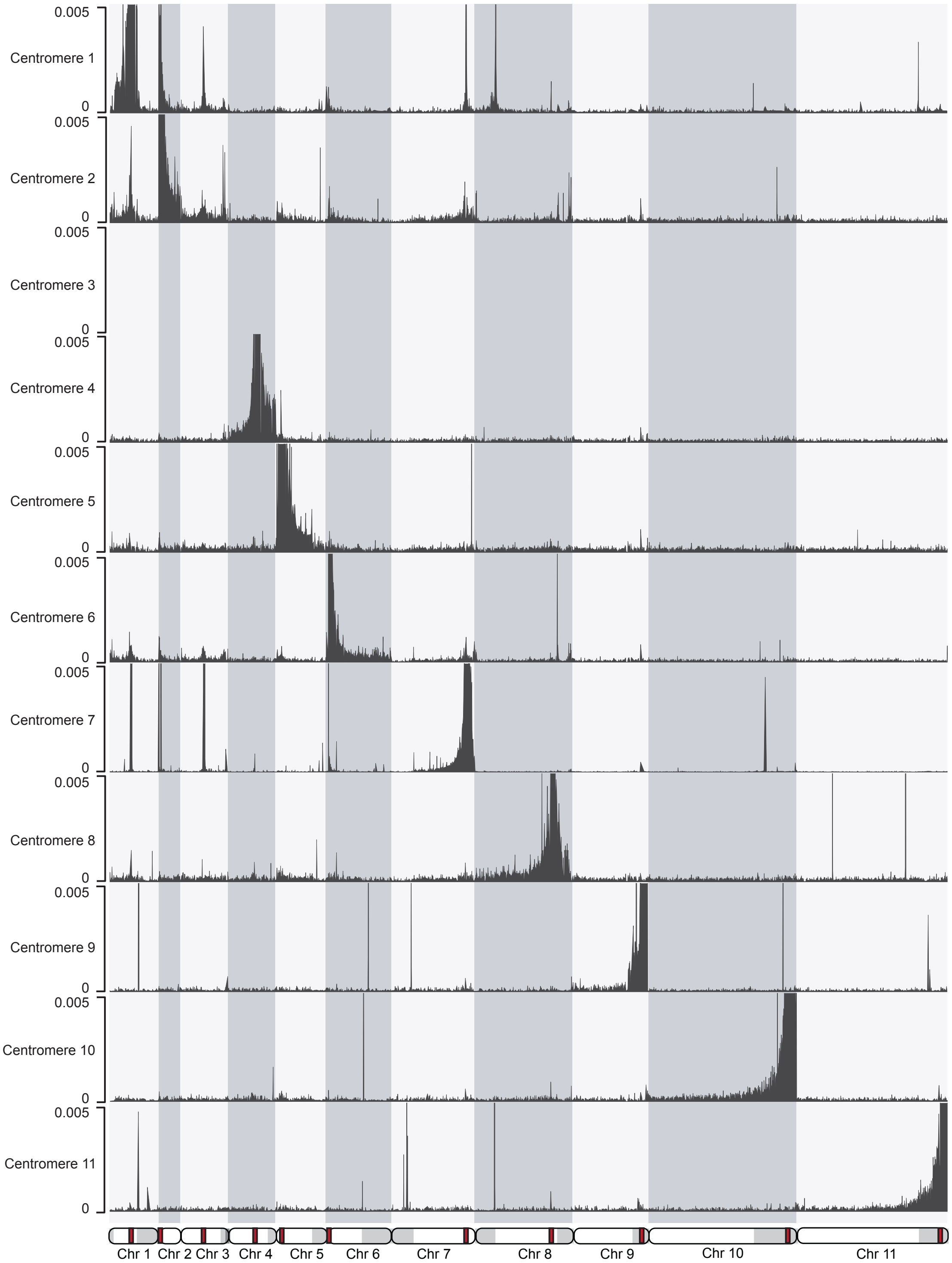

Supplementary Figure 10. Interaction frequencies of regions adjacent (downstream) to centromeres of haplotype B plotted against the entire genome, haplotype B. Virtual 4C interaction profiles based on Micro-C data (10 kb resolution) showing the interaction frequencies (y-axes) of 5 kb regions downstream the bins containing centromeres of genome B as points of view with the Tb427 genome version 12, haplotype B.
Bottom: each chromosome is displayed with the core region in white and subtelomeric regions in light gray. Centromeres are displayed as red boxes.

Spliced Leader repeats

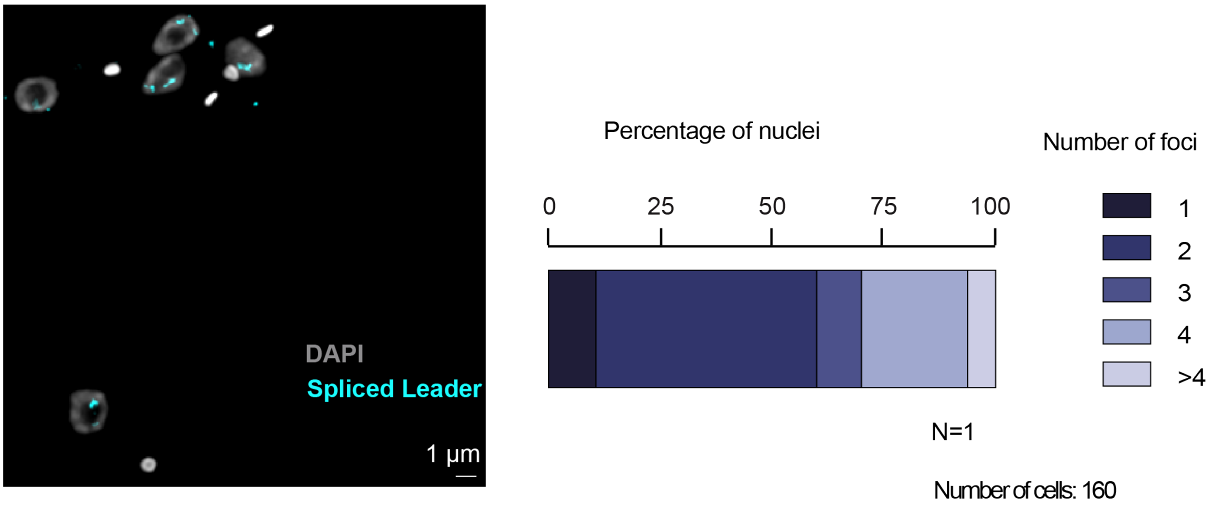

Supplementary Figure 11. FISH with probes designed against the Spliced Leader repeat locus reveals that the two alleles are not clustering. Fluorescence In Situ Hybridization (FISH) of the Spliced Leader (SL) repeats and quantification of SL foci (N=1; 160 nuclei). Cells displaying no signal were excluded. The SL locus is present in 2 copies that are not interacting in the nucleus.

References Supplementary Material

1. Benson, G. Tandem repeats finder: a program to analyze DNA sequences. *Nucleic Acids Res.* **27**, 573–580 (1999).
2. Cosentino, R. O., Brink, B. G. & Siegel, T. N. Allele-specific assembly of a eukaryotic genome corrects apparent frameshifts and reveals a lack of nonsense-mediated mRNA decay. *NAR Genomics Bioinforma.* **3**, lqab082 (2021).
3. Girasol, M. J. *et al.* RAD51-mediated R-loop formation acts to repair transcription-associated DNA breaks driving antigenic variation in *Trypanosoma brucei*. *Proc. Natl. Acad. Sci.* **120**, e2309306120 (2023).
4. Zimin, A. V. & Salzberg, S. L. The SAMBA tool uses long reads to improve the contiguity of genome assemblies. *PLOS Comput. Biol.* **18**, e1009860 (2022).
5. Yang, T. *et al.* HiCRep: assessing the reproducibility of Hi-C data using a stratum-adjusted correlation coefficient. *Genome Res.* **27**, 1939–1949 (2017).
6. Kruse, K., Hug, C. B. & Vaquerizas, J. M. FAN-C: a feature-rich framework for the analysis and visualisation of chromosome conformation capture data. *Genome Biol.* **21**, 303 (2020).
7. Wolff, J. *et al.* Galaxy HiCExplorer 3: a web server for reproducible Hi-C, capture Hi-C and single-cell Hi-C data analysis, quality control and visualization. *Nucleic Acids Res.* **48**, W177–W184 (2020).
